# Multi-Frequency-Scale Distributed Recurrence Plot-Based Fault Diagnosis for PMSM

**DOI:** 10.3390/s26041361

**Published:** 2026-02-20

**Authors:** Jun Sun, Ziling Nie, Yu Zhou, Pan Sun, Yangwei Zhou, Yihui Xia, Huayu Li

**Affiliations:** 1 Naval University of Engineering, Wuhan 430033, China; 1920191030@nue.edu.cn (J.S.);; 2 East Lake Laboratory, Wuhan 430079, China; 3 College of Electrical Engineering, Sichuan University, Chengdu 610065, China; 4 School of Electrical and Electronic Engineering, Huazhong University of Science and Technology, Wuhan 430074, China

**Keywords:** distributed recurrence plot (DRP), wavelet packet decomposition (WPD), multi-frequency scale, fault diagnosis

## Abstract

Conventional permanent magnet synchronous motor (PMSM) fault diagnosis methods rely on one-dimensional (1-D) time-series signals. These approaches face challenges such as complex signal processing, difficulty in extracting fault features, and limited noise immunity. To address these issues, a novel approach method is proposed. Its core process includes wavelet packet decomposition (WPD), distributed recurrence plot (DRP) generation, and image transformation. This approach enables feature representation of the original signal across multiple frequency bands, and the shortcomings of traditional recurrence plots in terms of feature redundancy and long-sequence representation are overcome. On this basis, a lightweight multi-frequency-scale fault diagnosis model is developed, consisting of a multi-frequency-scale convolutional neural network (CNN), a convolutional block attention module (CBAM), and a global average pooling (GAP) layer. Experimental results demonstrate that the proposed method achieves high diagnostic accuracy and strong noise immunity. Under identical hardware and dataset conditions, the inference time of the proposed method is only 12.35% as long as that of traditional recurrence plot-based CNN and 50.03% as long as that of asymmetric recurrence plot-based CNN.

## 1. Introduction

With the rapid electrification of transportation worldwide, permanent magnet synchronous motors (PMSMs) have become key components in electric vehicles and electric ships due to their high power density, high efficiency, and excellent dynamic performance [1]. However, various faults may still occur during actual operation, such as stator winding inter-turn short circuits, permanent magnet demagnetization, bearing wear, and rotor eccentricity [2,3]. In electric vehicles, such faults can directly affect overall safety, energy consumption, and driving comfort. In electric ships, where the operating environment is harsher and maintenance conditions are more constrained, the occurrence of these faults may lead to propulsion system failure and even severe navigation accidents [4]. Furthermore, accurate fault diagnosis is not only a prerequisite for the immediate maintenance of motor systems but also lays the foundation for their full life-cycle management. By identifying the performance degradation status of the motor, the system can further enable the prediction of Remaining Useful Life (RUL), thereby achieving the transition from “fault response” to “proactive prognostics”. Therefore, it is essential that fault diagnosis techniques for PMSMs be developed. Timely detection and classification of faults are crucial for ensuring the long-term safe and economical operation of electric drive systems [5].

Conventional motor fault diagnosis methods mainly rely on model-based analysis or signal processing techniques, such as motor current signature analysis, vibration signal analysis, and temperature monitoring [6,7]. In laboratory environments, these methods have achieved good performance and can identify faults accurately under specific operating conditions. However, in practical applications, their diagnostic performance is often severely restricted by varying operating environments, non-stationary operating conditions, environmental noise, and the complexity of motor control strategies. As a result, fault-related features are frequently masked by noise, which reduces the accuracy and reliability of fault diagnosis [8].

In recent years, with the rapid development of artificial intelligence, especially deep learning, new opportunities have emerged for motor fault diagnosis. It has been shown that converting one-dimensional (1-D) sensor time-series signals into two-dimensional (2-D) images can better reveal hidden patterns and significantly improve diagnostic accuracy, particularly under non-stationary and noise-contaminated operating conditions [9,10]. Moreover, convolutional neural networks (CNNs) exhibit marked advantages in processing image data, and their strong feature extraction capability makes them highly effective for complex fault signals. Consequently, transforming signals into image representations and employing CNNs for feature extraction and classification has become an important research direction in the field of motor fault diagnosis [11,12].

In the transformation of feature signals into images, various methods have been proposed. Examples include time–frequency images based on the continuous wavelet transform, Gramian angular summation/difference fields constructed via polar coordinate mapping, and symmetrized dot patterns obtained through coordinate mapping [13,14,15]. Among these approaches, recurrence plots (RPs) based on phase space reconstruction are regarded as particularly suitable for motor fault diagnosis because nonlinear characteristics can be effectively captured and the separability of fault patterns can be enhanced. However, recurrence plots generated by traditional methods still suffer from data redundancy, difficulties in representing long time series, large image sizes, and the masking of fault-related features in strongly noisy environments [16,17].

To alleviate these issues, several RP variants have been explored. Multi-scale (or signed) RPs construct recurrence representations at multiple resolutions to enrich texture details and improve discriminability across scales; however, they often increase computational overhead and may remain sensitive to embedding and threshold parameters [18,19]. Sliding-window RPs generate a sequence of local RPs from short-time segments to track non-stationary behaviors, but their performance strongly depends on manually tuned window length and stride, and continuous patterns may be fragmented across adjacent windows [20]. An RP combined with wavelet-based processing (e.g., wavelet compression) aims to reduce dimensionality and produce fixed-size images with lower storage and computation costs; nevertheless, fine recurrence textures can be blurred and the information loss is highly dependent on the chosen compression ratio [21].

Overall, although existing RP variants can improve diagnostic performance to some extent, they may not fully satisfy the practical requirements of PMSM diagnosis in terms of multi-frequency representation, compactness, and robustness and computational efficiency. On the one hand, it is desirable to explicitly characterize fault-related dynamics across different frequency bands; on the other hand, it is necessary to avoid the redundancy and computational burden caused by large symmetric recurrence matrices. Moreover, the method should reduce dependence on manually tuned hyperparameters such as window length and compression ratio, while maintaining stable recognition under noisy measurement conditions.

To this end, a lightweight fault diagnosis framework oriented toward multi-frequency dynamical characteristics is proposed, where wavelet packet decomposition (WPD), distributed recurrence plots (DRPs), and CNN-based recognition are integrated in a unified design. Different from conventional RPs and their windowed/compression-based variants, the measured leakage flux signal is first decomposed into multiple physically meaningful frequency sub-band components via WPD, and recurrence textures are constructed at the sub-band level to achieve a frequency-aware multi-scale representation. Then, a triangle-to-rectangle mapping is introduced to compactly reorganize the symmetric recurrence information. This mapping substantially reduces redundancy and yields a fixed-size representation. Furthermore, the recurrence textures from different sub-bands are structurally stacked and fed into a lightweight multi-branch multi-scale CNN, where convolutional block attention module (CBAM)-based attention is incorporated to enable adaptive cross-band fusion and noise suppression. With these designs, multi-frequency fault features can be more effectively exploited while maintaining low computational cost, thereby achieving accurate and robust fault identification.

The organizational structure of the remainder of this paper is as follows: Section 2 elaborates on the theoretical foundation for reconstructing 1-D motor leakage magnetic signals into RPs. Section 3 introduces the concept of DRPs, detailing the methods and steps for their establishment; building on this, it proposes a diagnostic method based on multi-frequency-scale CNNs using DRPs. Section 4 validates the effectiveness and superiority of the proposed diagnostic method through experiments and comparative analysis. Section 5 summarizes the research work presented in this paper.

## 2. Magnetic Leakage Flux Signal Reconstruction for PMSM

### 2.1. Phase Space Reconstruction of Magnetic Leakage Flux Signal

Chaos theory posits that the evolution of any variable in a system is determined by its interactions with other variables, thereby embedding information about those other variables in its developmental process [22]. For PMSM systems, if the magnetic flux leakage signal on the outer surface of the PMSM is measured, this signal represents the collective effect of all other relevant physical factors in the system, encompassing all information changes from all participating motions. Therefore, by extending the measured 1-D time series of the PMSM’s magnetic flux leakage signal to a higher-dimensional data space, all relevant information can be fully revealed. A common method to extend a 1-D time series to a higher-dimensional space is the phase space reconstruction method [10,23].

Assume that the measured magnetic flux leakage signal of the motor with length *N* is as shown in (1).

(1) $$x={\left[\;x(1),\;x(2),\;\ldots ,\;x(N)\;\right]}^{T},$$

By setting the delay time to τ and the embedding dimension to *m*, the reconstructed phase space is obtained as shown in (2).

(2) $$\left[\begin{array}{c} X(1)\\ X(2)\\ \vdots \\ X(N-(m-1)\tau ) \end{array}\right]=\left[\begin{array}{ccc} x(1) & \cdots & x(1+(m-1)\tau )\\ x(2) & \cdots & x(2+(m-1)\tau )\\ \vdots & \ddots & \vdots \\ x(N-(m-1)\tau ) & \cdots & x(N) \end{array}\right],$$

In (2), X(1),X(2),…,X(N−(m−1)τ) are points in the reconstructed *m*-dimensional phase space. From the above analysis, it can be seen that the delay time τ here actually refers to the number of sampling periods.

### 2.2. Determination of Embedding Dimension and Time Delay

From the steps of phase space reconstruction outlined in Section 2.1, it is evident that the selection of delay time τ and embedding dimension *m* significantly impacts the reconstructed phase space.

The choice of delay time directly influences the independence of the coordinate components in the reconstructed phase space. If the delay time τ is too short, the information correlation between adjacent coordinate components becomes high, making it difficult to effectively reveal the dynamic characteristics of the system. Conversely, if the delay time τ is too long, it may lead to the loss of critical information in the time series, thereby reducing the quality of the reconstructed phase space [24]. In this paper, the delay time τ is determined using the autocorrelation method.

First, the collected leakage magnetic field signal from the outer surface of the motor is processed to remove the mean value, resulting in the time series $x^{\prime }\left(n\right)$, as shown in (3):

(3) $$x^{\prime }\left(n\right)=x\left(n\right)-\frac{1}{N}\sum_{n=1}^{N}x(n),$$

Then, the autocorrelation function results are calculated at different delay times, denoted as $C_{xx}\left(\tau \right)$, as shown in (4):

(4) $$C_{xx}\left(\tau \right)=\sum_{n=1}^{N-\tau }x^{\prime }\left(n\right)x^{\prime }\left(n+\tau \right),$$

where τ is the given time delay. Finally, the normalized autocorrelation function result $\rho_{xx}\left(\tau \right)$ is calculated using (5):

(5) $$\rho_{xx}\left(\tau \right)=\frac{C_{xx}\left(\tau \right)}{\sigma_{x}^{2}},$$

where $\sigma_{x}^{2}$ is the variance of the original time series. Based on the definition in (3), the calculation formula is simplified as shown in (6).

(6) $$\sigma_{x}^{2}=\frac{1}{N}\sum_{n=1}^{N}{\left(x^{\prime }\left(n\right)\right)}^{2}.$$

When the normalized autocorrelation function $\rho_{xx}\left(\tau \right)$ first decreases to $\frac{1}{e}$, the corresponding time delay at this point is considered the optimal time delay $\tau_{op}$.

The embedding dimension *m* determines the dimensionality of the reconstructed phase space. If the embedding dimension is too low, it may fail to fully capture the system’s dynamic characteristics, resulting in information loss. Conversely, an excessively high embedding dimension, while potentially revealing more system complexity, increases computational cost and data requirements. In this paper, the false nearest neighbors method is employed to determine the embedding dimension.

Initially, a small embedding dimension *m* is selected. For m=1, the proportion of false neighbors is 100%. According to (7), the distance between each point and others in the constructed *m*-dimensional phase space is calculated, and the nearest neighbor for each point is identified.

(7) $$D_{m}\left(i,j\right)=∥X(i)-X(j)∥,(j\neq i)$$

In (7), $D_{m}\left(i,j\right)$ denotes the Euclidean distance between the *i*th and *j*th state vectors, X(i) and X(j), in the reconstructed *m*-dimensional phase space. The embedding dimension is then increased to (m+1), a new phase space is generated, and the corresponding distance $D_{m+1}\left(i,j\right)$ is evaluated. A neighbour X(j) is deemed a false nearest neighbour of X(i) if

(8) $$\frac{\left|\right|D_{m+1}\left(i,j\right)-D_{m}\left(i,j\right)\left|\right|}{D_{m}\left(i,j\right)}>r_{\mathrm{tol}}$$

where $r_{\mathrm{tol}}$ is a user-defined tolerance that quantifies the relative increase in distance caused by the additional embedding dimension.

The ratio of false neighbors to the total number of points in the *m*-dimensional phase space is calculated. The embedding dimension *m* is incremented in a stepwise manner, and the process is repeated. When the ratio falls below a predefined threshold, the current value of *m* is determined to be the minimum suitable embedding dimension.

## 3. Construction of DRP and Multi-Frequency-Scale Fault Diagnosis Algorithm

### 3.1. Wavelet Packet Decomposition of Magnetic Flux Leakage Signal

Wavelet packet decomposition (WPD) is an extension of the wavelet transform. It addresses the limitation of traditional wavelet decomposition, which recursively decomposes only low-frequency components, by enabling multi-frequency-scale decomposition of both high-frequency and low-frequency components. This facilitates more accurate signal analysis in the time–frequency domain.

The decomposition process for WPD can be expressed as follows:

(9) $$\left\{\begin{array}{c} c_{j+1,k}^{a}=\sum_{n}c_{j,n}^{a}h\left[n-2k\right]\\ c_{j+1,k}^{d}=\sum_{n}c_{j,n}^{d}g\left[n-2k\right] \end{array}\right.$$

where $c_{j,k}^{a}$ and $c_{j,k}^{d}$ represent the approximation and detail coefficients at layer *j*, respectively.

**Figure 1:**
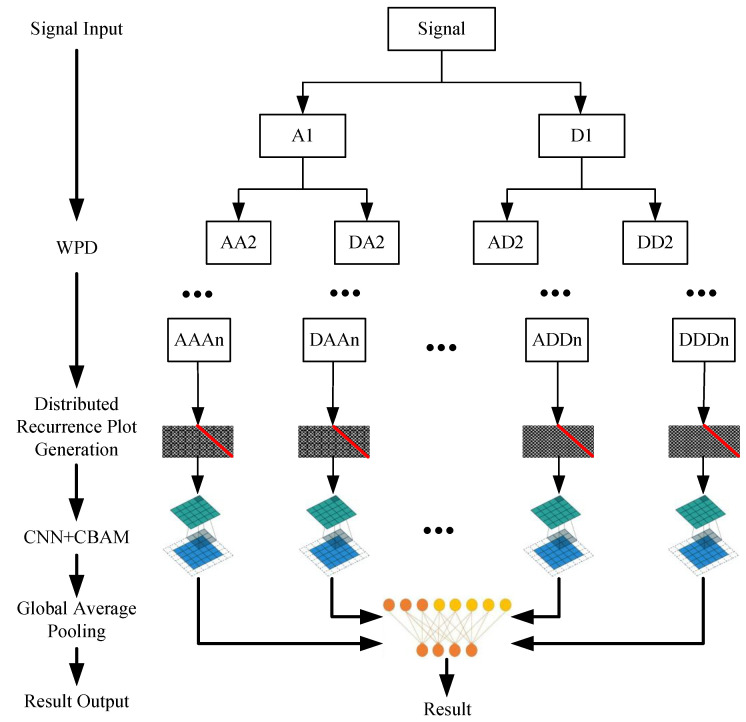
Roadmap of multi-frequency-scale fault diagnosis technology based on DRP. The vertical flow on the left outlines the main processing stages. In the central architecture, white boxes represent the hierarchical decomposition of the signal via Wavelet Packet Decomposition (WPD). Gray textures with red diagonal lines denote the Distributed Recurrence Plot (DRP) generation for each sub-band. Teal and blue layered blocks indicate the CNN+CBAM modules for feature extraction, while the orange/yellow nodes represent the Global Average Pooling and fusion process for the final result output. Black arrows indicate the direction of data flow and the decomposition hierarchy.

The WPD algorithm achieves a full-domain decomposition of the signal, with its decomposition process exhibiting a binary tree structure, as illustrated in Figure 1. The original magnetic leakage signal is processed through a pair of mirror filters, namely a low-pass filter ($h\left[n-2k\right]$ in (9)) and a high-pass filter ($g\left[n-2k\right]$ in (9)), resulting in the generation of two sub-bands: the low-frequency approximation coefficients (A1 in Figure 1) and the high-frequency detail coefficients (D1 in Figure 1). During the second level of decomposition, the same filtering operation is applied simultaneously to both the first-level low-frequency approximation coefficients (A1) and high-frequency detail coefficients (D1). This process yields four sub-bands: the low-frequency approximation coefficients (AA2 in Figure 1) and high-frequency detail coefficients (DA2 in Figure 1) derived from the first-level low-frequency approximation coefficients (A1), and the low-frequency approximation coefficients (AD2 in Figure 1) and high-frequency detail coefficients (DD2 in Figure 1) derived from the first-level high-frequency detail coefficients (D1). This decomposition process extends iteratively in a similar manner, ultimately forming a complete binary tree structure. At each decomposition level, WPD synchronously decomposes the low-frequency and high-frequency coefficients from the preceding level, thereby enabling the extraction of information features from the original signal across multiple time and frequency scales.

For a magnetic flux leakage signal time series of length *N*, after *J* levels of WPD, the number of sub-bands is $2^{J}$, producing $2^{J}$ frequency bands. This enables feature extraction of the magnetic flux leakage signal across these $2^{J}$ frequency bands. When down-sampling is applied and the length of the wavelet basis function is neglected, the length of each sub-band is $L_{J}$, given by

(10) $$L_{J}=\frac{N}{2^{J}}$$

where *J* is the decomposition level.

### 3.2. The Construction of DRPs

From the analysis in Section 2, a sub-band of length $L_{J}$ can be reconstructed into a new phase space with embedding dimension *m* and time delay $\tau_{op}$, yielding a distance matrix $D_{m}\left(i,j\right)$ of all point pairs in this phase space.

The distance matrix is normalized to the range [0,1] using (11) as follows:

(11) $${\tilde{D}}_{m}\left(i,j\right)=\frac{D_{m}\left(i,j\right)-min\left(D_{m}\left(i,j\right)\right)}{max\left(D_{m}\left(i,j\right)\right)-min\left(D_{m}\left(i,j\right)\right)}$$

where $max(D_{m}\left(i,j\right))$ and $min(D_{m}\left(i,j\right))$ represent the maximum and minimum values of the distance matrix, respectively, and ${\tilde{D}}_{m}\left(i,j\right)$ denotes the normalized distance between the *i*-th and *j*-th points in the *m*-dimensional phase space.

The normalized distance matrix is then used to generate grayscale values for the RP, with distance values directly mapped to grayscale intensity. This process produces $2^{J}$ RPs, each with a resolution given by

(12) $$\left[\frac{N}{2^{J}}-\left(m-1\right)\tau_{op}\right]\times \left[\frac{N}{2^{J}}-\left(m-1\right)\tau_{op}\right]$$

**Figure 2:**
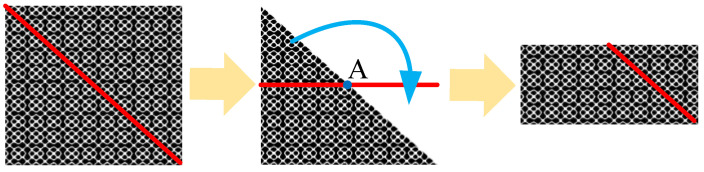
Proposed image transformation process. The red line represents the main diagonal (Line of Identity) of the recurrence plot. The blue curved arrow illustrates the geometric mapping or rotation strategy used to transform the lower triangular matrix into a compact rectangular format. The yellow arrows indicate the sequential steps of the transformation workflow.

Based on the above analysis, RPs exhibit symmetry along the main diagonal, with ${\tilde{D}}_{m}\left(i,j\right)={\tilde{D}}_{m}\left(j,i\right)$, leading to redundant features. To reduce computational complexity, each of the $2^{J}$ RPs can be divided along the main diagonal, forming isosceles right triangles. Further dividing these triangles at the midpoint of the hypotenuse results in smaller isosceles triangles and quadrilateral regions. Rotating the smaller triangles 180° clockwise around a predefined point (the midpoint of the hypotenuse, denoted as point A in Figure 2) transforms the regions into rectangles with a resolution specified in (13).

(13) $$\left[\frac{N}{2^{J}}-\left(m-1\right)\tau_{op}\right]\times \left[\frac{N}{2^{J+1}}-\frac{\left(m-1\right)\tau_{op}}{2}\right]$$

As illustrated in Figure 1 and Figure 2, the original 1-D signal undergoes WPD, phase space reconstruction, RP generation, and image transformation. These processes yield a set of RPs with uniform resolution. Each RP corresponds to information from a specific frequency band. In this paper, these RPs are collectively referred to as DRPs.

When down-sampling is applied and the number of WPD layers increases, the lengths of the generated approximation and detail coefficient sequences correspondingly decrease, satisfying the condition

(14) $$\sum_{2^{J}}\left[\frac{N}{2^{J}}-\left(m-1\right)\tau_{op}\right]\leq N$$

The DRPs decompose the original signal into recurrence images across different frequency bands, providing a multi-frequency-scale graphical representation of PMSM health and addressing the long-time-scale characterization limitations of traditional plots. During DRP transformation, the symmetric and redundant portions of the plots are removed, mitigating the data redundancy issue inherent in traditional RPs.

From (13), as the number of decomposition layers increases, the number of pixels in each DRP image decreases. Specifically, the total pixel count across all DRP images in a given layer is approximately 0.5 times that of the preceding layer. Consequently, increasing the decomposition level leads to a gradual reduction in the computational cost of the fault diagnosis algorithm.

### 3.3. Multi-Frequency-Scale CNN Fault Diagnosis Algorithm

CNNs are effective for image-based fault classification due to their ability to perform local feature extraction, weight sharing, and translation invariance [25]. This subsection proposes a CNN-based fault diagnosis model using DRPs for a PMSM at multiple frequency scales.

**Figure 3:**
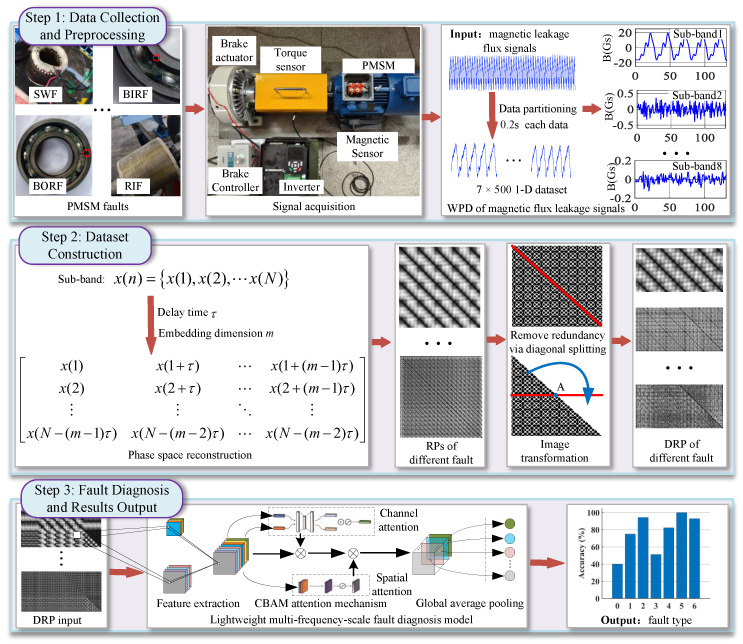
Fault diagnosis flowchart of the proposed method. The workflow consists of three main stages: Step 1 illustrates the data collection platform and signal preprocessing; Step 2 demonstrates the dataset construction process, including phase space reconstruction and the generation of Distributed Recurrence Plots (DRP) via diagonal splitting to remove redundancy; Step 3 displays the lightweight fault diagnosis model architecture based on CNN and CBAM attention mechanisms. The red arrows indicate the sequential flow between the main processing stages.

Regarding the system workflow, the proposed fault diagnosis framework consists of three stages, as illustrated in Figure 3:

Stage 1: Data Collection and Preprocessing. The original magnetic flux leakage signals of the PMSM are collected via magnetic sensors and partitioned into 0.2 s sample units. Subsequently, WPD is employed to decompose each sample into $2^{J}$ sub-band sequences across different frequency ranges, providing a foundation for multi-scale feature extraction.

Stage 2: DRP Dataset Construction. Phase space reconstruction is performed for each sub-sequence, with the delay time $\tau_{op}$ and embedding dimension *m* determined by the autocorrelation method and false nearest neighbors method, respectively. Through an image transformation mechanism (diagonal splitting, rotation, and splicing), the original symmetric recurrence plots are converted into redundancy-free rectangular DRP stacks, effectively addressing feature redundancy and long-sequence representation issues.

Stage 3: Fault Diagnosis and Result Output. The DRP stacks are fed into a lightweight DRP-MCNN method. The model employs $2^{J}$ parallel input branches to extract multi-scale features and integrates the CBAM to adaptively reinforce key features while suppressing environmental noise. Finally, global average pooling (GAP) is utilized to reduce computational complexity, and the recognition results for seven motor health conditions are accurately delivered through a Softmax layer.

**Figure 4:**
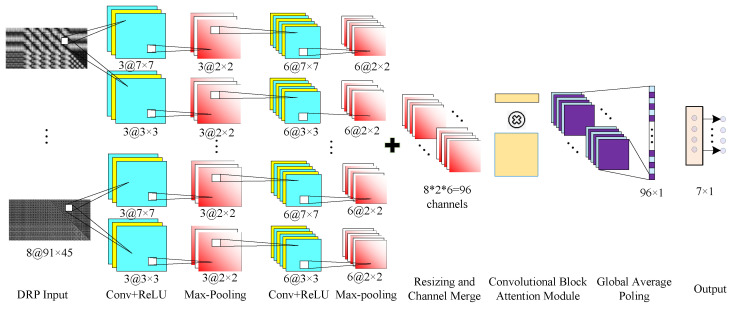
The proposed multi-frequency-scale PMSM fault diagnosis method based on DRPs. The cyan blocks represent the convolutional layers with ReLU activation, and the red blocks denote the max-pooling layers. The yellow and purple blocks illustrate the internal components of the Convolutional Block Attention Module (CBAM). The symbol “+” indicates the resizing and channel merging operation that fuses features from different branches, while the symbol “×” represents element-wise multiplication for applying attention weights.

The complete fault diagnosis model and network configuration are shown in Figure 4. Two different convolution kernel sizes (7×7 and 3×3) are used for multi-scale feature extraction on each branch’s input. After the input passes through two convolutional layers (each followed by a pooling layer), a total of 96 feature channels are obtained. Due to the different kernel sizes, these 96 channels have inconsistent spatial dimensions. To address this, the smaller feature maps are padded with zeros to match the size of the larger ones. All channels are then concatenated and fed into the CBAM attention mechanism, resulting in 96 refined feature channels.

In the fault diagnosis method proposed here, the use of a multi-scale CNN enables the extraction of both local details and global contextual information from the plots, thereby enhancing the model’s feature extraction capacity, minimizing feature loss, and ultimately improving diagnostic accuracy.

The convolution kernels across individual branches are randomly initialized and mutually independent, endowing the model with an inherent degree of noise robustness. Moreover, the CBAM module allows the network to emphasize key features along both the channel and spatial dimensions, further improving noise immunity. To facilitate lightweight deployment, global average pooling is employed for feature fusion, transforming the 96 channels into a 96×1 feature vector and minimizing computational cost.

## 4. Experiments and Verification

### 4.1. Description of the Fault Experiment Platform

**Figure 5:**
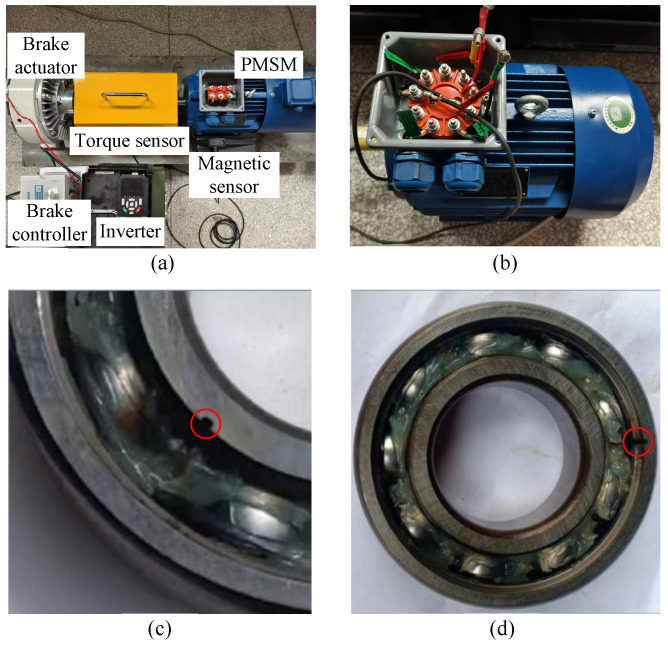
Experimental platform and partial motor fault schematic diagram. (**a**) Experimental platform. (**b**) Inter-turn short-circuited PMSM. (**c**) Bearing with inner race fault. (**d**) Bearing with outer race fault.

The motor used in this paper is a 5.5 kW, four-pole-pair, three-phase interior PMSM. Specific motor and experimental parameters are provided in Table 1. The experimental platform consists of five main parts: (1) PMSM, (2) torque sensor, (3) inverter, (4) magnetic field sensor, and (5) brake and brake controller. The experimental platform and some faulty PMSMs are shown in Figure 5.

This paper collects magnetic flux leakage data from PMSMs under seven distinct conditions: normal operation (NOF), stator winding fault (SWF), bearing with inner race fault (BIRF), bearing with outer race fault (BORF), local demagnetization fault (LDF), uniform demagnetization fault (UDF), and rotor imbalance fault (RIF). The fault configurations were implemented as follows:

SWF: A tap was drawn from phase A of the motor winding and short-circuited, creating an imbalance in the three-phase ABC windings. Specifically, the number of turns in phase A was reduced by 3 turns compared to the other two phases, with each normal phase winding consisting of 46 × 6 turns.

BIRF: The motor bearing consists of an inner race, outer race, rolling elements, and cage. The inner race fault was simulated by machining a groove of 1 mm width and 1.5 mm depth on the inner race. At the rated speed, the characteristic frequency of the bearing inner race fault is 135.8 Hz.

BORF: Similarly, the outer race fault was simulated by machining a groove of 1.0 mm width and 1.5 mm depth on the outer race. At the rated speed, the characteristic frequency of the bearing outer race fault is 89.2 Hz.

LDF: A local demagnetization fault was simulated by removing one permanent magnet from the motor, subjecting it to high-temperature demagnetization, and reinstalling it.

UDF: A uniform demagnetization fault was simulated by replacing all permanent magnets in the motor with magnets of reduced magnetic strength.

RIF: An imbalance fault was created by adding weight patches to the rotor, resulting in an imbalance of 15 g.

### 4.2. Construction of a PMSM Health Characteristic Dataset

This paper employs an AMS-2K AC magnetic field sensor to measure the magnetic flux leakage signal of a PMSM. Magnetic flux leakage signals are collected from the motor’s surface under both no-load and rated-speed conditions for various motor health states, including normal operation and multiple fault types. The collection point is located about 1 cm from the PMSM side, parallel to the axis, and the signals are sampled at a rate of 5 kHz. The collected signals are subsequently decomposed using WPD and converted into DRPs to form a dataset.

Each dataset unit comprises 0.2 s of data (1000 samples), yielding 3500 non-overlapping units across multiple motor health conditions, with 500 units per fault type. Random sampling is applied to select 400 units from each fault type dataset for training and 100 units for testing. Table 2 presents the motor labels and the corresponding dataset unit counts.

### 4.3. Comparative Analysis of the Proposed Method with Other Fault Diagnosis Methods

**Figure 6:**
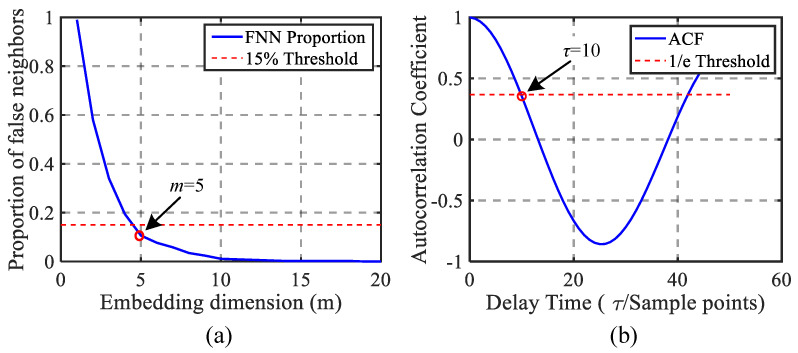
Selection of delay time and embedding dimension. (**a**) Trend in false neighbors proportion with embedding dimension. (**b**) Trend in autocorrelation coefficient with delay time.

As detailed in Section 2.2, the autocorrelation method is employed to determine the delay time. Figure 6a illustrates the variation in the autocorrelation function with respect to the delay time. For this analysis, the parameter $r_{tol}$ is set to 0.4, resulting in a delay time of 10 sampling cycles.

Subsequently, the embedding dimension is determined using the false nearest neighbors method, based on the established delay time. Figure 6b depicts the variation in the percentage of false neighbors as a function of the embedding dimension. The minimum embedding dimension is selected when the proportion of false neighbors decreases to 15%, resulting in an embedding dimension *m* of 5.

In constructing the DRP, this paper employs the db4 wavelet and conducts WPD up to three decomposition levels (*J* = 3), resulting in $2^{J}=8$ sub-bands and, consequently, eight modified recurrence images in the plot. Thus, the proposed method comprises eight parallel CNN branches. The proposed method is termed the distributed recurrence plot multi-frequency-scale convolutional neural network (DRP-MCNN).

To demonstrate the superiority of the proposed DRP-MCNN method, two main comparative experiments are designed as follows:

1. Traditional Recurrence Plot-based CNN Method (TRP-CNN): The magnetic flux leakage signals of the PMSM undergo direct phase space reconstruction to produce a large recurrence plot for fault diagnosis [17].
2. Asymmetric Recurrence Plot-based CNN Method (ARP-CNN): The original magnetic flux leakage signal is divided into two segments. RPs are generated separately, and their upper-triangular parts are extracted based on symmetry. These parts are subsequently combined into an asymmetric recurrence plot for fault diagnosis [26].

It is noteworthy that the number of convolutional and pooling layers, as well as the sizes of the convolution and pooling kernels, are identical across the proposed and comparative methods. Specifically, a network with five hidden layers is designed, comprising two convolutional layers and two max-pooling layers. For each input branch, the convolutional kernels adopt sizes of 7 × 7 and 3 × 3, respectively. The first convolutional layer yields three outputs, while the second produces six. All max-pooling layers employ a 3 × 3 window with a stride of 2. The training process spans 20 epochs with a learning rate of 0.001. The network structure is depicted in Figure 4. Training and image processing are performed using an Intel i7-11600H CPU, an NVIDIA RTX3050 GPU, and 16 GB RAM.

Table 3 presents a comparison of the image generation, training, and validation times across different fault diagnosis methods. The image generation time of the DRP-MCNN method is comparable to that of ARP-CNN and substantially shorter than that of TRP-CNN. This efficiency stems from the reduced data length in DRP-MCNN during WPD, which accelerates recurrence plot generation. Despite additional image processing steps, the time required by DRP-MCNN remains similar to that of ARP-CNN. In contrast, TRP-CNN employs the full-length original signal without preprocessing, resulting in the longest image generation time due to its higher computational demand, as elaborated in Section 2.2.

Regarding model computational complexity, Table 3 shows the total time required for training and validation by each diagnostic method over 20 epochs. The proposed DRP-MCNN method achieves the shortest fault diagnosis runtime: on the training set, its time cost is only 15.83% of that of TRP-CNN and 51.05% of that of ARP-CNN; on the test set, it requires only 12.35% of the TRP-CNN time and 50.03% of the ARP-CNN time. The proposed method requires only 70.5 ms for single-sample processing. When combined with the 0.2 s acquisition window, the total latency is less than 0.3 s, which can satisfy real-time detection requirements. This advantage is primarily attributed to the down-sampling applied during WPD of the raw magnetic flux leakage signal in the proposed method. The cumulative length of all sub-bands post-decomposition is less than that of the original signal. As a result, the pixel count of images across the eight branches is lower than that of images generated by ARP-CNN and TRP-CNN, leading to reduced computational effort during fault diagnosis.This finding indicates that incorporating WPD into the fault diagnosis pipeline can substantially reduce the model’s computational time.

**Figure 7:**
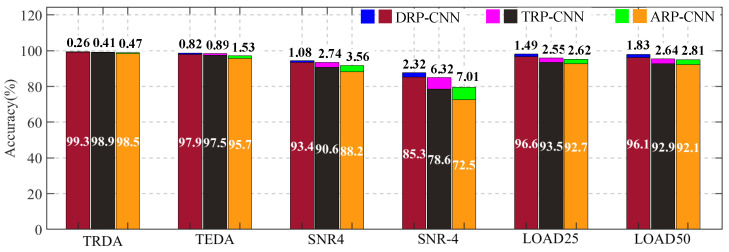
Comparison of diagnostic accuracy of different fault diagnosis methods under various conditions.

To mitigate the randomness inherent in the fault diagnosis process, this paper employed *k*-fold cross-validation to verify the repeatability of all trained models. Specifically, five-fold cross-validation was adopted, with the accuracy results and corresponding standard deviation illustrated in Figure 7. In this figure, “TRDA” denotes the diagnostic accuracy comparison on the training set, while “TEDA” represents the diagnostic accuracy comparison on the test set. Each large rectangular bar corresponds to the diagnostic accuracy, and the smaller bar atop each large bar indicates the standard deviation derived from the five-fold cross-validation. As observed from the figure, the DRP-MCNN, ARP-CNN, and TRP-CNN methods all achieve diagnostic accuracies exceeding 98% on the training set. On the test set, the method proposed in this paper demonstrates a higher average accuracy of 97.9% [95%CI: 96.9–98.9%], accompanied by a notably smaller standard deviation of 0.82%. This is followed by the TRP-CNN method, with a diagnostic accuracy of 97.5% [95%CI: 96.4–98.6%], while the ARP-CNN method exhibits the least favorable performance, achieving an accuracy of 95.7 ± 1.53% [95%CI: 93.8–97.6%]. These results underscore the superior diagnostic accuracy and stability of the proposed method.

**Figure 8:**
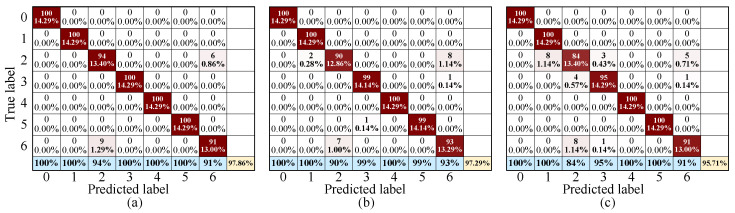
Confusion matrix comparison of different fault diagnosis methods on the test set. (**a**) Proposed DRP-MCNN methods. (**b**) TRP-CNN. (**c**) ARP-CNN.

The confusion matrix provides an intuitive representation of the distribution of correct and incorrect predictions in classification results, serving as an effective tool for evaluating the classification performance of fault diagnosis methods. Figure 8 presents a comparative analysis of the confusion matrices for various fault diagnosis methods on the test set (displaying a representative result from the five-fold cross-validation). In the figure, the vertical axis represents the true labels of the data, while the horizontal axis represents the predicted labels obtained through the fault diagnosis algorithms. Consequently, the numbers in each square along the diagonal indicate the quantity of correctly classified data and their percentage of the total dataset. The last row shows the percentage of correctly classified data relative to each fault type’s dataset, with the final number on the diagonal representing the overall diagnostic accuracy of the fault diagnosis algorithm. As observed from the figure, the proposed method achieves a diagnostic accuracy of 97.8% on the presented set. Specifically, in terms of per-class metrics, it attains 100% precision and recall for normal motors, winding faults, bearing outer race faults, local demagnetization faults, and uniform demagnetization faults. For the more challenging bearing inner race faults and rotor imbalance faults, both precision and recall remain above 91%. The diagnostic results of the proposed DRP-MCNN method outperform those of the TRP-CNN and ARP-CNN methods. These results demonstrate that the proposed DRP-MCNN method exhibits superior recognition performance across different fault types compared to the other methods.

**Figure 9:**
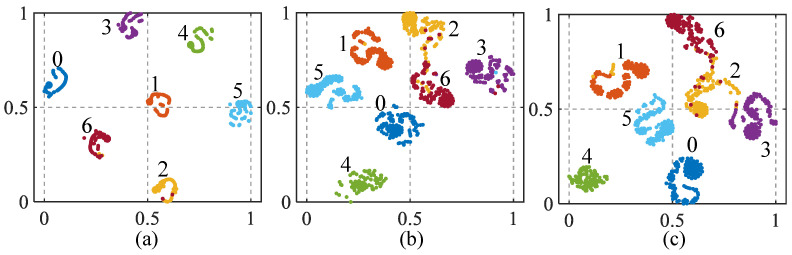
Comparison of t-SNE visualization results for the diagnostic performance of different fault diagnosis algorithms. (**a**) Proposed DRP-MCNN method. (**b**) TRP-CNN. (**c**) ARP-CNN.

To intuitively compare the fault classification performance of the proposed method, t-distributed stochastic neighbor embedding (t-SNE) is employed to map high-dimensional features from the trained model to a two-dimensional plane. Figure 9 presents the dimensionality reduction results for 700 test set samples. Different colors and numbers denote distinct fault types and their corresponding labels.

From Figure 9, the DRP-MCNN method demonstrates effective inter-class separability, characterized by significant separation between different fault classes. In contrast, the TRP-CNN and ARP-CNN methods exhibit notable inter-class overlaps and reduced separation, suggesting less effective feature representation. This underscores the superior fault feature representation of the DRP-MCNN method, primarily due to the effective integration of WPD, the DRP, and the CNN, which collectively enhance feature extraction and diagnostic accuracy.

**Figure 10:**
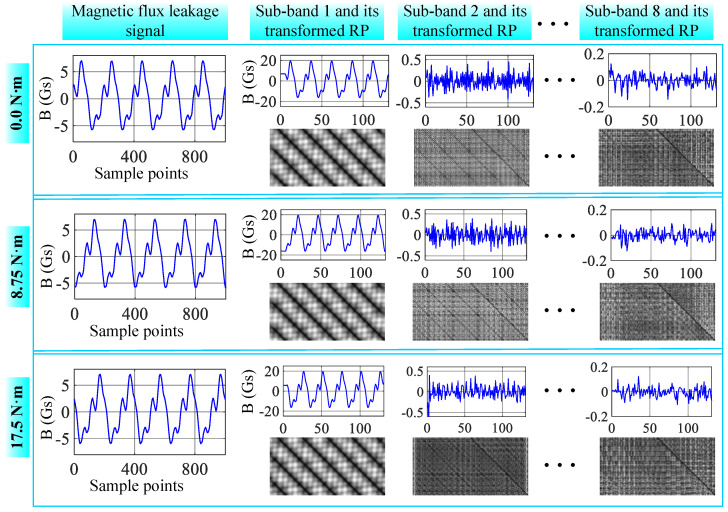
Magnetic flux leakage signal under different loads and its sub-bands after WPD and DRPs.

Figure 10 presents the magnetic flux leakage signal, WPD, and DRP of a PMSM with local demagnetization faults under different load conditions. It can be observed from the figure that the load has little impact on the PMSM’s magnetic flux leakage signal, and thus the load has minimal effect on the diagnostic accuracy. Figure 10 shows the PMSM fault diagnostic accuracy when applying the pre-trained model to conditions of 25% rated load (LOAD25 in Figure 10, 8.75 N·m) and 50% rated load (LOAD50 in Figure 10, 17.5 N·m). Compared to the no-load condition (TEDA in Figure 10, 0 N·m), the diagnostic accuracy shows little change, though the standard deviation slightly increases. Notably, the DRP-MCNN method proposed in this paper exhibits the smallest change, demonstrating stronger adaptability to different operating conditions.

As indicated by the preceding analysis, in a noise-free environment, the method proposed in this paper demonstrates superior diagnostic performance compared to other fault diagnosis methods. To further validate the fault diagnosis capability of the proposed algorithm under noisy conditions, Gaussian noise with signal-to-noise ratios (SNRs) of 4 dB and −4 dB was manually injected into the collected PMSM magnetic flux leakage signals, thereby constructing new datasets with varying noise intensities. Figure 10 presents the average diagnostic accuracy and standard deviation of three different fault diagnosis algorithms when tested on PMSM magnetic flux leakage signals with different SNRs. In this figure, “SNR4” denotes the diagnosis with Gaussian noise at an SNR of 4 dB, while “SNR-4” represents the diagnosis with Gaussian noise at an SNR of −4 dB. As observed from the figure, even under strong noise conditions, the proposed method maintains a fault diagnosis accuracy of 85.3 ± 2.32% [95%CI: 82.4–88.2%]. Furthermore, across all noise levels, the fault diagnosis results of the proposed DRP-MCNN method outperform those of the other comparative methods, fully verifying the effectiveness and rationality of the proposed feature graph construction approach.

The experimental results demonstrate that the method proposed in this paper exhibits exceptional diagnostic performance and noise resistance. This outcome is primarily attributed to two key factors: (1) WPD: WPD decomposes the magnetic flux leakage signal, extracting noisy signals across different frequency bands, which significantly enhances the fault diagnosis model’s anti-interference capability. (2) MCNN: The multi-frequency-scale CNN integrated with the CBAM module enables adaptive weight assignment along the two principal dimensions—channel and spatial—thereby allowing the network during training to accentuate salient features and, in turn, minimize the influence of noise and other non-salient features on the diagnostic outcome.

### 4.4. Ablation Study on the Multi-Frequency-Scale Method

To further comprehensively assess the role of the CBAM module within the proposed multi-frequency-scale fault diagnosis model and to verify the soundness of the feature fusion strategy, two sets of ablation experiments were designed in the diagnostic model. The experimental settings and results are presented below.

1. **Effectiveness of the CBAM module:** Using the diagnostic model shown in Figure 4 as the baseline, we removed the CBAM module while keeping all other components unchanged and trained and tested the model on the same dataset. The model without CBAM achieved a training set diagnostic accuracy of 99.1 ± 0.33% and a test set accuracy of 97.1 ± 1.39% [95%CI: 95.4–98.8%]; completing 20 epochs required 217.42 s, and the testing phase required 37.01 s. By comparison, the proposed full model (with CBAM) achieved an accuracy of 97.9 ± 0.82%, yielding a performance gain of ΔAccuracy = +0.8% and a stability improvement of ΔStdev = −0.57%; the parameter count increased by only 675 (from 5.13 k to 5.80 k), and the computational cost FLOPs increased by 0.025 M (from 7.475 M to 7.5 M). Although introducing CBAM incurs additional computational overhead (the total test time increased by 0.50 s relative to the model without CBAM), this overhead is acceptable in engineering practice.
2. **Comparison between global average pooling and fully connected layers:** To further reduce algorithmic complexity, this study employs global average pooling in place of conventional fully connected layers for feature aggregation. For a fair comparison, all other components of the network were kept unchanged and global average pooling was replaced with fully connected layers. The fully connected-based variant achieved training and test set diagnostic accuracies of 99.4 ± 0.25% and 98.0 ± 0.77% [95%CI: 97.0–99.0%], respectively, which are nearly identical to those obtained with global average pooling-based aggregation. However, the training time for 20 epochs increased to 229.71 s and the test time to 38.45 s, i.e., increases of 8.15 s and 0.94 s, respectively. Therefore, adopting global average pooling preserves diagnostic accuracy while reducing algorithmic complexity, thereby facilitating lightweight deployment of the model.
3. **Decoupling analysis of component contributions:** To explicitly distinguish the independent contributions of WPD and the triangular-to-rectangular geometric transformation, we conducted controlled experiments while maintaining an identical network backbone. First, to evaluate the necessity of frequency decomposition, we replaced WPD with a windowed RP strategy, where the signal was split into eight non-overlapping segments to generate eight corresponding RPs. The results showed a significant drop in accuracy to 93.2 ± 3.01% [95%CI: 89.46–96.94%] (compared to 97.9 ± 0.82% for the proposed method), demonstrating that multi-frequency-scale features offer superior fault discrimination compared to simple time-domain slices. Second, to assess the geometric transformation, we retained the WPD but removed the reshaping step, instead utilizing symmetry to zero-pad the redundant triangular region while keeping the original square dimensions. While this baseline maintained a comparable accuracy of 97.8 ± 0.80% [95%CI: 96.81–98.79%], the larger input pixel count resulted in a total inference time of 70.28 s for 20 epochs. In contrast, the proposed method required only 37.51 s, improving computational efficiency by approximately 47.14%. These results confirm that the WPD decomposition guarantees high diagnostic precision, while the triangular-to-rectangular transformation significantly reduces computational overhead without compromising accuracy.
4. **Hyperparameter selection (sensitivity of *J*):** We systematically evaluated the impact of the wavelet packet decomposition level J∈{2,3,4} on model performance through comparative experiments. The results indicate that setting J=2 leads to a significant reduction in test accuracy to 93.7 ± 1.44% [95% CI: 91.91–95.49%], primarily due to insufficient frequency resolution for effectively separating fault characteristics. Furthermore, the lower decomposition level results in longer signal lengths per sub-band, which increases the total pixel count of the generated recurrence plots and extends the inference time to 74.36 s. Conversely, increasing *J* to 4 reduces the inference time due to feature dimension compression; however, the diagnostic accuracy drops by 2.3% (to approximately 95.6%) compared to the J=3 baseline, accompanied by an increased standard deviation, which indicates weakened model stability. In contrast, J=3 achieves the highest diagnostic precision (97.9% [95% CI: 96.88–98.92%]) while maintaining a reasonable computational cost (37.51 s). Therefore, J=3 is selected as the optimal operating point to balance diagnostic accuracy and computational efficiency.
5. **Impact of input resolution:** Since the proposed method inherently reduces pixel redundancy via geometric transformation, its input dimensions are significantly smaller than those of conventional RPs. To rule out the possibility that the performance differences are solely attributable to resolution disparities, we resized the inputs of the baseline methods (TRP-CNN and ARP-CNN) to match the total pixel count of the DRP input (8×91×45=181×181). The results indicate that under this iso-resolution condition, the computational costs for the baselines decreased substantially: the training times for 20 epochs dropped to 214.41 s and 215.29 s and the testing times to 33.06 s and 35.85 s, respectively, becoming comparable to or even slightly lower than the proposed DRP-MCNN. However, this efficiency gain came at the cost of a significant degradation in diagnostic performance. The accuracy of TRP-CNN dropped to 85.63 ± 6.85% [95% CI: 77.12–94.14%] and ARP-CNN to 89.02 ± 4.38% [95% CI: 83.58–94.46%]. In contrast, DRP-MCNN maintained superior diagnostic performance under the same resolution constraint. This confirms that the advantage of the proposed method lies in its high-density feature representation, which preserves critical frequency-domain fault features even at reduced image resolutions.
6. **Robustness against data leakage:** To ensure rigorous evaluation and eliminate potential temporal correlation leakage, we implemented a “Leave-Group-Out” (session-wise) splitting protocol. Specifically, the training and testing sets were derived from distinct experimental trials to guarantee independence, while strictly adhering to a 4:1 split ratio. Under this strict protocol, the model achieved an accuracy of 97.7 ± 0.79% [95% CI: 96.76–98.72%], which is statistically consistent with the random split result (99.4%). This confirms that the model learns robust fault features rather than memorizing temporal artifacts from specific recording sessions.
7. **Generalization across operating speeds:** To evaluate the proposed method’s adaptability to variable working conditions, we conducted cross-speed validation experiments. The model was trained solely on data collected at the rated speed (1500 rpm, no load) and tested directly on unseen data at 1125 rpm (75% rated speed) and 750 rpm (50% rated speed). The results demonstrated strong generalization: the proposed method maintained a diagnostic accuracy of 89.5 ± 4.65% at 1125 rpm and 87.0 ± 5.68% at 750 rpm. In contrast, the baseline methods (TRP-CNN and ARP-CNN) exhibited significant performance degradation under these variable-speed conditions, with accuracies dropping by over 20% compared to the rated-speed benchmark. This evidence confirms that the WPD-DRP features possess strong invariance against frequency shifts caused by speed variations.

### 4.5. Generalization Validation on Public Datasets

To further substantiate the generalizability of the proposed fault diagnosis approach, an external validation was conducted on a publicly available PMSM dataset released by the Human Laboratory, Department of Mechanical Engineering [27]. The dataset was measured in accordance with the ISO 10816-1:1995 [28] international standard. The measurement conditions are summarized in Table 4. A benchmark dataset comprising six health conditions was constructed, including inter-turn short circuits (severity levels: 0.0%, 6.48%, and 21.69%) and coil-to-coil short circuits (severity levels: 1.01%, 3.93%, and 7.56%). Each sample spans four electrical periods (1710 points). For each condition, 500 samples were collected and randomly split into 400 for training and 100 for testing, with no temporal overlap between samples. The experimental results are reported in Table 5.

As summarized in the table, the proposed DRP-MCNN method achieves the shortest runtime (303.77 s for training and 59.54 s for testing), followed by ARP-CNN (810.36 s/159.91 s), whereas TRP-CNN is the most time-consuming (3401.39 s/614.72 s). The training time of the proposed method is only 8.93% of that of TRP-CNN and 37.49% of that of ARP-CNN, reaffirming the efficiency advantage of distributed recurrence plots for fault diagnosis.

From the diagnostic results, the proposed DRP-MCNN method achieves the highest test accuracy of 99.61 ± 0.20% [95%CI: 99.36–99.86%], exceeding TRP-CNN (97.78 ± 4.78% [95%CI: 91.90–100.00%]) and ARP-CNN (97.18 ± 4.91% [95%CI: 91.08–100.00%]) by 1.83% and 2.43%, respectively. Furthermore, the standard deviation of the DRP-MCNN results is significantly smaller (±0.20%), further corroborating the effectiveness and robustness of the proposed DRP-MCNN method approach.

## 5. Conclusions

A new graphical representation method for 1-D time-series signals, referred to as the DRP method, is proposed. The core procedure of this method includes WPD, RP generation, and image transformation. Using this approach, signal features are extracted across multiple frequency bands. The feature redundancy problem of traditional RPs is resolved, and the difficulty in representing long-time-series signals is alleviated.

Based on this approach, a lightweight multi-frequency-scale fault diagnosis model named DRP-CNN was developed. The model is composed of a multi-frequency-scale CNN, a CBAM attention module, and a global average pooling layer. Compared to the existing ARP-CNN and TRP-CNN methods, the proposed DRP-MCNN method achieves higher diagnostic accuracy and stronger noise robustness. Under identical hardware and dataset conditions, its inference time is only 12.35% of that of TRP-CNN and 50.03% of that of ARP-CNN. Given its low parameter count and operational efficiency, the proposed method is well-suited for deployment in resource-constrained scenarios, such as embedded monitoring systems and edge-based motor diagnostics. Moreover, the non-invasive nature of the magnetic flux sensing approach enhances its engineering applicability. By prioritizing the topological features of the phase space over absolute signal amplitudes, the proposed method exhibits theoretical robustness against amplitude variations caused by sensor positioning errors or thermal drift, thereby facilitating robust industrial deployment.

Future work will focus on extending the current framework to diagnose compound faults under variable working conditions and exploring few-shot learning to reduce the reliance on fully labeled datasets.

The main contributions of this paper are as follows:

1. Multi-Frequency-Scale Signal Representation: A novel method for representing 1-D time-series signals as multi-frequency-scale images is proposed. This method eliminates the feature redundancy present in traditional RPs and alleviates the difficulty of representing long-time-series signals.
2. WPD in Fault Diagnosis: WPD is applied to graphical fault diagnosis, enabling fault features to be represented at multiple frequency scales. As a result, the computational complexity of converting 1-D signals into 2-D images and extracting features is significantly reduced.
3. Lightweight Multi-frequency-scale CNN Fault Diagnosis: A lightweight multi-frequency-scale CNN-based fault diagnosis method is developed, achieving high diagnostic accuracy, strong robustness to noise, and low computational cost. Under the same hardware and dataset conditions, the inference time of the proposed method is only 12.35% of that of TRP-CNN and 50.03% of that of ARP-CNN.

## Figures and Tables

**Table 1:** Motor and experimental parameters.

Parameters	Value	Parameters	Value
Motor power	5.5 kW	Rated frequency	100 Hz
Rated voltage	380 V	Rated speed	1500 rpm
Motor poles	8	Sampling frequency	5 kHz
Rated torque	35 N·m	Rotor structure	Interior

**Table 2:** Dataset size and labels for motors in different health conditions.

Fault Type	Label	Number of Training Sets	Number of Testing Sets
NOF	0	400	100
SWF	1	400	100
BIRF	2	400	100
BORF	3	400	100
LDF	4	400	100
UDF	5	400	100
RIF	6	400	100

**Table 3:** Time cost comparison of different fault diagnosis methods.

	Proposed DRP-MCNN Method	TRP-CNN	ARP-CNN
IG500	21.56 s	184.13 s	30.84 s
IP500	12.35 s	/	11.06 s
TR20	221.56 s	1399.44 s	434.03 s
TE20	37.51 s	303.61 s	74.98 s

Note: IG500: Time required to generate 500 images. IP500: Time required to process 500 images. TR20: Time required for training with 20 epochs. TE20: Time required for testing with 20 epochs.

**Table 4:** Experimental parameters of the public dataset.

Parameters	Value	Parameters	Value
Motor power	1.0 kW	Rated frequency	60 Hz
Motor poles	4	Rated speed	3000 rpm
Rated torque	3.18 N·m	Sampling frequency	25.6 kHz

**Table 5:** Comparison of results for different fault diagnosis methods on the public dataset.

	Proposed DRP-MCNN Method	TRP-CNN	ARP-CNN
IG500	34.15 s	103.96 s	55.95 s
IP500	13.96 s	/	12.79 s
TR20	303.77 s	3401.39 s	810.36 s
TE20	59.54 s	614.72 s	159.91 s
Training Accuracy	99.82 ± 0.05%	98.10 ± 2.35%	97.92 ± 2.10%
Test Accuracy	99.61 ± 0.20%	97.78 ± 4.78%	97.18 ± 4.91%

## Data Availability

Publicly available datasets were analyzed in this study. This data can be found here: Ref. [27].
